# Iron Metabolism in the Recovery Phase of Critical Illness with a Focus on Sepsis

**DOI:** 10.3390/ijms25137004

**Published:** 2024-06-26

**Authors:** Xiyang Zhang, Bruce Holbein, Juan Zhou, Christian Lehmann

**Affiliations:** 1 Department of Anesthesia, Pain Management and Perioperative Medicine, Dalhousie University, Halifax, NS B3H 1X5, Canada; zhangxiyang17@gmail.com (X.Z.); juan.zhou@dal.ca (J.Z.); 2 Guangdong Provincial Key Laboratory of Precision Anaesthesia and Perioperative Organ Protection, Department of Anesthesiology, Nanfang Hospital, Southern Medical University, Guangzhou 510515, China; 3 Department of Microbiology & Immunology, Dalhousie University, Halifax, NS B3H 1X5, Canada; beholbein@sympatico.ca; 4 Department of Physiology & Biophysics, Dalhousie University, Halifax, NS B3H 1X5, Canada; 5 Department of Pharmacology, Dalhousie University, Halifax, NS B3H 4R2, Canada

**Keywords:** critical illness, iron deficiency, recovery, iron supplementation

## Abstract

Iron is an essential nutrient for humans and microbes, such as bacteria. Iron deficiency commonly occurs in critically ill patients, but supplementary iron therapy is not considered during the acute phase of critical illness since it increases iron availability for invading microbes and oxidative stress. However, persistent iron deficiency in the recovery phase is harmful and has potential adverse outcomes such as cognitive dysfunction, fatigue, and cardiopulmonary dysfunction. Therefore, it is important to treat iron deficiency quickly and efficiently. This article reviews current knowledge about iron-related biomarkers in critical illness with a focus on patients with sepsis, and provides possible criteria to guide decision-making for iron supplementation in the recovery phase of those patients.

## 1. Introduction

Critical illness includes medical conditions involving vital organ dysfunction and resulting in a high risk of imminent death and the requirement of intensive care [1]. Critical illness results in characteristic changes to iron metabolism. Up to 75% of critically ill patients show elevated ferritin but low serum iron levels at admission to the intensive care unit (ICU) [2]. Most critically ill COVID-19 patients showed significantly decreased levels of serum iron during the first 24 hours of their ICU stay [3]. Moreover, as the most common nutritional deficiency worldwide, iron deficiency, including absolute iron deficiency (AID) and functional iron deficiency (FID), represents a risk factor for developing critical illness [2,4]. Generally, AID is characterized by reduced iron stores and inadequate iron supply; whereas, FID means that iron stores are adequate, but iron supply is insufficient [5]. The combination of pre-existing disturbances of iron metabolism and regulatory changes in iron metabolism in critical illness represents a diagnostic dilemma for the management of iron metabolism in critically ill patients. In general, iron replacement therapy is not considered during the acute phase of critical illness because more accessible iron is beneficial for invading microbes and oxidative stress [2,6]. However, during the recovery phase of critical illness, clinicians are facing the question of when to (re)start iron supplementation. This review summarizes the existing knowledge of the changes in iron metabolism during critical illness as the basis for deciding on iron supplementation in the recovery phase of critical illness.

## 2. The Role of Iron in Human Health

Iron plays an important role in human health (for review see [7]). It is necessary for the transport of oxygen, mitochondrial function, and the synthesis of deoxyribonucleic acid. Iron is also involved in immune regulation since its role in the Fenton reaction, which is required for the formation of oxygen-free radicals (OFR), a main inflammatory mediator [8]. Levels of iron must be tightly regulated because of the potentially detrimental effects of iron overload. Iron uptake is under metabolic control by various regulators such as hepcidin [9]. Surprisingly, humans do not have an active iron excretion pathway. However, under inflammatory conditions, through the hepcidin/ferroportin axis and the iron-regulatory protein/iron-responsive elements (IRP/IRE) system, organisms struggle to maintain iron homeostasis by reducing the uptake and shifting iron to the intracellular space [10]. Therefore, therapeutic iron supplementation needs to consider the dynamics of iron homeostasis.

## 3. Iron Metabolism in Critically Ill Patients

Critical illness is accompanied by characteristic changes in iron metabolism, with microcytic anemia as the leading clinical symptom [11,12,13]. FID without anemia is also common in critical illness. FID, measured by red cell hypochromasia on flow cytometry and presence of zinc protoporphyrin, represents a situation of insufficient iron availability but adequate iron storage [14,15]. In a prospective observational study, it was reported that the incidence of FID reached up to 35% in patients at admission [4]. In a cohort of 314 patients from an interdisciplinary ICU, about 28.3% of patients were diagnosed with FID [16]. Moreover, considering iron metabolism, the presence of severe inflammation is discriminant to understand the pathophysiological basis of anemia. Most investigators concurred that during the early stage of critical illness with sepsis, due to the release of pro-inflammatory mediators, hepcidin levels are up-regulated, which disturbs the balance of iron recycling [2,17]. For example, increased hepcidin negatively regulates the activity of ferroportin in macrophages and hepatocytes, which decreases the ability of ferroportin to export iron from cells into the circulation, and this results in the insufficient availability of transferrin-bound iron for erythropoiesis.

An increasing body of evidence has shown that the levels of iron play a vital role in the outcome of critical illness with sepsis. A prospective single-center study demonstrated that iron and serum transferrin saturation (TSAT) levels are strong predictors of outcome in ICU patients [18]. Interestingly, iron and TSAT levels were significantly decreased in sepsis survivors, and transferrin levels were lower in non-survivors [18]. Potential reasons for higher serum iron levels in non-survivors were reviewed previously [18]. Insufficient activation of hepcidin is considered as one of the main reasons. Severe sepsis is often accompanied by liver dysfunction, which reduces hepatic hepcidin synthesis [19]. Secondly, non-survivors show increased catabolism, with iron being released from the elevated turnover of erythrocytes and other cells [20,21]. Thirdly, to improve tissue perfusion, frequent blood transfusions will further increase iron levels from aged and damaged erythrocytes. Therefore, serum iron levels can also be used as a marker of disease severity and prognosis [22]. In a clinical study with COVID-19 patients, the authors demonstrated that serum iron levels were significantly decreased in patients with mild respiratory failure (RF) compared to those without RF, but there were no significant differences in iron levels between the non-RF and severe RF groups, so there is a U-shaped relationship between serum iron levels and disease severity [23]. Another study including a total of 1,891 patients with sepsis showed that higher serum iron levels were associated with increased 90-day mortality [24]. 

On the other hand, non-transferrin-bound iron (NTBI) may be more meaningful than conventional serum iron levels for forecasting adverse clinical outcomes and in-hospital mortality [25]. NTBI includes different sub-pools of circulating iron that are not safely bound to transferrin. NTBI sub-pools can accelerate the Fenton reaction to generate reactive oxygen species (ROS), increase oxidative stress, cause tissue damage, and increase the risk of mortality in critical care patients [26,27]. Lele and coworkers demonstrated a positive association between NTBI levels and mortality in patients with acute coronary syndrome, similar to sepsis [28]. However, NTBI levels must be interpreted in conjunction with transferrin saturation. In the study cited above [25], the authors measured bleomycin reactive iron in the serum of patients with transferrin saturation below 20%. This situation might not be comparable with conditions where transferrin saturation is high and/or transferrin production is disturbed (see Section 4.3). Also, pre-existing iron deficiency has a significant impact on the pathogenesis of critical illness, and has to be considered for the interpretation of iron levels as acute biomarkers. 

## 4. Iron Metabolism in the Recovery Phase from Critical Illness

### 4.1. Ferritin

Ferritin is a protein devoted to the storage of intracellular iron, but it is also present in the bloodstream [29]. Ferritin levels are used to evaluate iron storage in the body. Ferritin is also used as an indicator by the body to assess iron status in infection, inflammation, and malignancy. For example, a recent systematic review reported that the ferritin level in serum was considered as a diagnostic marker for iron storage, with ferritin concentration in patients with iron depletion as low as 80 µg/L, while a ferritin level of nearly 500 µg/L was defined as iron overload [30]. Recently, serum ferritin has also been used for assessing iron metabolism and organ dysfunction in patients with sepsis. In a prospective study of critical care patients, non-survivors showed a higher increase of serum ferritin than survivors, and the serum ferritin level was positively correlated with the Sequential Organ Failure Assessment (SOFA) score [31]. It was suggested that increased serum ferritin levels were linked to inflammation [31,32]. Since serum ferritin could be metabolized and excreted through urine, measuring urinary ferritin was suggested as a potential non-invasive screening test for iron metabolism in neonatal intensive care unit (NICU) patients [33]. To detect iron-limited erythropoiesis, the amended urine ferritin was found to have very high sensitivity and specificity, and the positive predictive value was 100%.

**Figure 1:**
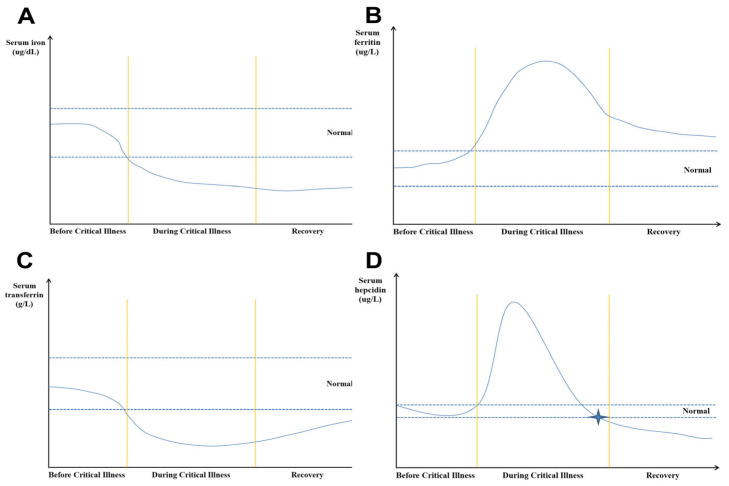
Summary of the iron metabolism-related markers dynamics in critical illness: (**A**) changes in serum iron; (**B**) changes in serum ferritin; (**C**) changes in serum transferrin; (**D**) changes in serum hepcidin. 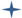 represents the potential starting point for iron supplementation.

The levels of serum ferritin were found to be significantly associated with the severity of disease and mortality in hospitalized COVID-19 patients. Increased levels of serum ferritin were present in patients with more severe disease [34,35,36,37,38] and with a higher mortality rate [39]. Increased levels of serum ferritin may enhance the inflammatory response, so as to exert a pathogenic role in viral infection [40]. In addition, excessive ferritin can increase ROS production and enhance oxidative stress [41,42,43]. Furthermore, associations of hyperferritinemia with a worse prognosis of disease were also reported in both acute and post-acute phases [34,44,45,46]. Therefore, during the recovery phase of ICU patients, serum ferritin is still an important marker of iron metabolism. If the serum ferritin level continues to be high, it indicates that the inflammatory response of the body has not been fully controlled, and even if the serum iron concentration is low, it is not the time to supplement iron. Based on the information above, we summarized the serum-iron-with-ferritin dynamics in surviving ICU patients with a long ICU stay in Figure 1.

### 4.2. Hepcidin

Hepcidin is the master iron regulatory hormone, which is synthesized in the liver, circulates in the blood stream, and is excreted in the urine. Hepcidin regulates intestinally absorbed, macrophage-recycled or liver-stored iron efflux from cells into the blood circulation through the regulation of the expression of ferroportin on cell surfaces. It is reported that hepcidin is positively associated with ferritin, interleukin-6, C-reactive protein, and red blood cell transfusion. Hepcidin is also negatively associated with levels of iron and transferrin, total iron binding capacity, and reticulocyte response [47]. In a prospective single-center clinical non-interventional study, which included one hundred adult surgical ICU patients, the authors found that the levels of hepcidin in serum showed a time-dependent course (Figure 1D). The serum levels of hepcidin were significantly increased upon ICU admission, and markedly decreased during the ICU stay [47]. During the recovery phase in ICU, most of the patients’ reticulocyte responses were increased, and their inflammatory responses were diminished. Therefore, the hepcidin concentrations decreased. This suggests that measurements of hepcidin levels may be useful for managing iron deficiency in patients with sepsis during the recovery phase [48]. In fact, AID was defined when hepcidin was below 20 µg/L, whereas hepcidin levels from 20 µg/L to 41 µg/L were defined as FID [49]. However, many different factors must be considered for decision-making since hepcidin levels can be abnormal before critical illness, e.g., in patients with pre-existing liver and renal diseases, heart failure, or who are on steroid therapy.

In addition, serum hepcidin can predict the mortality of patients in ICU during the recovery phase. Hepcidin was suggested as a clinical biomarker for the severity of COVID, since the levels of serum hepcidin were significantly increased in ICU non-surviving patients, compared with ICU survivors. This indicates that, in septic patients admitted to ICU, high levels of hepcidin are positively associated with mortality [50]. Also, some studies showed that, the higher the baseline levels of serum hepcidin in hospitalized patients with COVID-19, the higher the possibility of mechanical ventilation and kidney replacement for these patients [36,50]. However, Yağcı et al. reported that ICU patients with COVID-19 showed lower levels of serum hepcidin than healthy patients did [35]. It was speculated there was a similar structure between the cytoplasmic tail of SARS-CoV-2-spiked glycoprotein and hepcidin protein, so SARS-CoV-2 directly enhances the levels of tissue and circulating ferritin, induces deficiency of hemoglobin and serum iron, and suppresses hepatic hepcidin synthesis [35]. Moreover, in a prospective multicenter study including 2087 patients in 28 ICUs, the authors used plasma hepcidin levels to diagnose AID and assess the association of AID with outcomes during the recovery phase after an ICU stay. They showed that AID diagnosed on the basis of a hepcidin level lower than 20 ng/l at ICU discharge was correlated with higher one-year mortality, and a hepcidin level lower than 10 ng/l showed poor one-year physical recovery [48]. Therefore, to accelerate the speed of physical recovery, during the recovery phase of septic patients dynamic monitoring of the levels of hepcidin might be used to guide the timeline in starting iron supplementation.

### 4.3. Transferrin and Transferrin Saturation

As the primary iron transport protein, transferrin binds iron and delivers iron into the cells of target organs and tissues via receptor-mediated endocytosis. Since transferrin can stand without binding iron, transferrin saturation (TSAT) reflects the amount of iron bound to transferrin, and is used as a reliable marker of systemic iron status [29]. Recent findings demonstrated that parameters of iron metabolism, particularly transferrin and TSAT, can be utilized as strong outcome predictors for diverse groups of patients with sepsis [18]. Brandtner et al. investigated the impact of serum iron-related parameters on the outcome of sepsis, and showed that low transferrin concentrations and higher TSAT levels were associated with reduced survival [51]. Therefore, they suggested that TSAT can serve as a stand-alone predictor for sepsis survival and improve the prediction power of the SOFA score [51].

Similar to the case of septic patients, evidence has demonstrated the association of transferrin (or TSAT) with disease severity in patients with liver dysfunction. It was reported that iron metabolism was disrupted in patients with acute-on-chronic liver failure (ACLF), and showed that high TSAT and low transferrin levels related to the severity of ACLF and short-term mortality [52,53]. A similar correlation between TSAT and disease severity was also reported in decompensated cirrhosis patients [54]. Different from the case of septic patients, the release of transferrin from hepatocytes in patients with ACLF is inhibited, and decreased serum transferrin prevented the recovery of patients. One reason is that low levels of serum transferrin can cause the formation of NTBI, which promotes oxidative stress and endothelia injury. On the other hand, as one of the important factors affecting the recovery of critically ill patients, infection can be increased by the elevated availability of iron levels, which are induced by decreased serum levels of transferrin.

The relationship of transferrin levels and TSAT with severity of disease was also reported in COVID-19 patients. For example, low transferrin levels were found at the time of their hospitalization, especially in patients with serious medical conditions such as high oxygen demand, and their decreased levels of transferrin were even more intense [29]. Inpatients and critically ill patients showed significantly lower levels of transferrin than outpatients, indicating that low levels of transferrin can predict enhanced inflammation and increased disease severity [55]. Similarly, TSAT in COVID patients with severe respiratory failure was markedly higher than in patients with mild or no respiratory failure. For example, higher TSAT was present in intubated patients rather than non-intubated patients [23,35]. Moreover, the levels of TSAT in COVID-19 patients showed a dynamic pattern. Generally, in the early stage of infection, such as at ICU admission, the levels of TSAT were not very high but increased between the 3^rd^ and 6^th^ day of admission. Then, in the later stage, generally within 7–18 days of staying in the hospital, their levels returned to normal [56]. Therefore, because the change of transferrin is mainly regulated by the availability of iron, during the recovery phase of COVID-19 patients the levels of transferrin, especially TSAT, can be used as an important reference to guide the timing of iron supplementation. The serum transferrin dynamics in critically ill patients with long ICU stays is shown in Figure 1C.

### 4.4. Iron Supplementation

In the recovery phase from critical illness with sepsis, iron supplementation should be considered for septic patients once the diagnosis of FID is established. Appropriate iron supplementation is safe and beneficial to the recovery of these patients. Giving intravenous iron to patients without FID increases the risk of toxic side effects and iron overload [57].

Iron deficiency and anemia are frequent and may impair recovery in septic patients. High-dose intravenous iron can be administered safely under the guidance of hepcidin levels. During the recovery phase of critically ill patients with sepsis, if the serum hepcidin level is below 20 μg/L, it may be an appropriate time to start iron supplementation (Figure 1D).

## 5. Harm of Dysregulated Iron Metabolism in the Recovery Phase of Critical Illness

Despite protective effects during the critical phase of critical illness, low iron levels in the recovery phase of critically ill patients are harmful [20,21,22,23,24]. This is because, during the process of iron recycling, reticuloendothelial cells such as macrophages, can phagocytose and decompose the aging or damaged erythrocytes, then release iron back into circulation. Therefore, if the iron is sequestered in reticuloendothelial cells, and it is difficult to release it back into the cycle, the recovery of critically ill patients will be affected. For example, hypoferremia may reflect a detrimental intracellular iron overload during inflammatory processes and impair oxygen delivery to peripheral tissues by limiting erythropoiesis [24,26].

### 5.1. Anemia

Anemia is a common condition in critically ill patients in ICU. It is present in up to 65% of all patients at the time of admission, and in nearly 97% after a stay of eight days in ICU [16]. Anemia is correlated with worse outcomes, including prolonged stay and increased mortality. The etiology of anemia in critically ill patients is often multifactorial and complex, influenced by underlying chronic diseases and complicated by relevant blood loss, such as for diagnostic investigations, hemolysis or surgery. However, the most common reason for anemia in hospitalized patients is iron-restricted erythropoiesis, which is usually caused by FID [58]. During the recovery phase of critical illness, reduced uptake of nutritional iron from intestinal cells and retention of iron into the reticulohistiocytic system (RHS) are the characteristic features of iron-deficiency anemia [16,59]. Early clinical trials have demonstrated that iron supplementation can elevate serum transferrin and increase erythropoiesis in critically ill patients, so transfusion of red blood cells and supplementation with iron are the common therapeutic strategies [60]. Recently, Litton et al. also reported that combined supplementation of iron and erythropoietin could promote the recovery of intensive care patients with anemia (hemoglobin < 100 g/L) [61]. Conversely, a multicenter randomized trial in trauma patients found that iron supplementation markedly raised the serum level of ferritin without affecting the levels of transferrin saturation, hemoglobin levels, and iron-deficient erythropoiesis, and there was no requirement for packed red blood cells (RBC) transfusion [62]. There are several reasons for the discrepancies of these studies. First, different iron agents were used in the trials. Second, baseline differences between the iron group and the placebo group were observed in the last trial, despite randomization.

### 5.2. Cognitive Dysfunction

Cognitive dysfunction is often present in survivors of critical illness, affecting more than 25% of patients and often persisting after physical recovery [63]. 

For example, cognitive and functional status was assessed in 821 patients with respiratory failure or shock at 3 and 12 months after discharge. The results showed that, among survivors of critical illness at three months post-discharge, about 26% to 40% of patients with traumatic brain injury or Alzheimer’s disease suffered from cognitive dysfunction. And those levels persisted for 12 months for some of these patients [64]. Although the pathophysiology of cognitive dysfunction after critical illness is multifactorial, and the exact mechanism is not very clear and needs more investigation, iron deficiency is considered an important contributing factor. It was reported that iron deficiency is related to poor health and severe neurological impairment, such as emotional and neurocognitive dysfunction [65]. The mechanisms of how iron deficiency affects behavior include changes in the hippocampus, the corpus striatum, certain neurotransmitters, redox balance, and myelination [66]. Especially in metabolically active brain tissue, iron plays an important role in neurotransmitter synthesis and metabolism, and it is necessary to maintain mitochondrial function [67]. In line with dopaminergic dysfunction, it is considered that iron deficiency can affect our cognitive abilities [68]. Although evidence shows that iron supplementation contributes to improving quality-of-life and cognitive function for iron-deficient patients [64,65], the effects of iron supplementation on cognitive outcomes in patients recovering from critical illness need further study, especially the optimal dose and timing.

### 5.3. Fatigue

Fatigue is a common symptom in critically ill patients. For example, a high prevalence of fatigue was reported in many populations of ICU survivors at a 12-month follow-up after medical, elective, and urgent surgery [69]. In addition, critical illness, weakness or myopathy is frequently persistent in many patients experiencing reduced exercise capacity and quality of life, even years after the acute event [70]. Among the complex variety of causes, it was reported that FID has been associated with muscular weakness, fatigue, and impaired post-ICU rehabilitation [71]. In an observational study among cardiac surgery patients, the score of physical fatigue in FID patients on day 7 was higher than non-iron deficiency patients [72]. However, some animal studies and clinical reports indicated that the relationship between iron deficiency and fatigue seems independent of anemia [73,74,75]. For example, Zhou et al. revealed that, through positively regulating skeletal muscle-specific mitochondrial biogenesis and energy production, long-term iron supplementation in combination with vitamin B6 led to less body weight gained and increased maximal oxygen uptake in rats [76]. Therefore, further investigation of the relationship between critical illness fatigue and iron deficiency may allow us to better understand the role of iron supplementation in promoting physical recovery following acute severe illness. Furthermore, because physiological iron uptake is very low (about 1 to 2 mg/day), correction of iron deficiency after an ICU stay should be prolonged [1].

### 5.4. Cardiopulmonary Dysfunction

It is well known that iron is a co-factor for many enzyme activities in vital cellular and organismal functions; in particular, iron is very important for some high-energy-demand cells, such as cardiomyocytes, hepatocytes, and skeletal cells [77]. Because iron is required for the activity of prolyl hydroxylase that leads to hypoxia-inducible factor (HIF) degradation, iron deficiency induces the expression of HIF. Therefore, iron is involved in the systemic response to hypoxia and plays an important role in the cardiopulmonary recovery of critically ill patients. 

Independent of anemia, AID is associated with an increased risk of death in patients with heart failure [78]. Although the underlying causes of AID in heart failure are poorly characterized, several factors have been recognized as contributing to the high prevalence of AID in heart failure, including advanced age, female gender, chronic inflammation, kidney failure, malnutrition, and reduced iron absorption, as well as increased iron loss [77]. Recently, Mordi et al. demonstrated that clinical outcomes of chronic heart failure in non-anemic iron-deficient patients were worse than in anemic iron-replete patients, suggesting that iron deficiency can affect heart failure directly and in a way different from the effects of anemia [79]. Results from animal experiments have shown that iron homeostasis of cardiomyocytes is closely related to the severity of heart failure, e.g., in a mouse model, absence of cardiomyocyte transferrin receptor 1 (TfR1) significantly reduced the level of iron in cardiomyocytes, resulting in fatal heart failure. This is due in part to the failure of mitochondrial respiration [80]. Upregulation of hepcidin and downregulation of myocardial TfR1 have been proposed as the mechanisms involved in the critical-illness-induced impairment of cardiac iron availability [81]. Importantly, evidence has suggested that treatment with intravenous iron for patients who have systolic heart failure significantly improved exercise capacity, quality of life, and survival rate [82]. However, high-dose oral iron treatment in patients with heart failure and AID did not show improvement in exercise capacity [83]. Therefore, further investigation is needed to confirm the effectiveness of iron supplementation in the rehabilitation of heart failure patients with AID.

Iron deficiency is also present in 33–46% of patients with pulmonary artery hypertension (PAH), often in patients admitted to the ICU with severe acute respiratory distress syndrome [84]. PAH is often associated with a reduction in exercise capacity and oxygen handling, deterioration of right ventricular function, and even mortality [85]. Both oral and intravenous iron supplementation provided better clinical outcomes in patients with PAH [84]. In a retrospective study, researchers analyzed the long-term effects of iron supplementation with ferric carboxymaltose (FCM) on iron status and clinical parameters in patients with PAH and iron deficiency. The results showed a significant improvement in exercise capacity and World Health Organization functional class. Cases of hospitalization for worsening PAH over 12 months were also significantly reduced [86].

## 6. Potential Therapeutics for Iron Deficiency in the Recovery Phase from Critical Illness

Due to its effectiveness, safety, and low cost, oral iron, such as ferrous sulfate, is the first-line treatment for most patients with iron deficiency. However, the oral-drug-delivery route presents several disadvantages in critically ill patients, such as bad absorption of iron or adverse gastrointestinal reactions [87]. In addition, because of the inhibiting effect of high hepcidin levels on intestinal iron absorption, oral iron supplementation in critically ill patients is not efficient. Moreover, intravenous iron supplementation can accomplish a quick replenishment of a large deficiency. Therefore, intravenous iron is commonly used in clinical medicine to reduce the risks and limitations related to oral iron. Several intravenous irons are being used, such as iron sucrose (IS), iron gluconate (IG), iron dextran, iron ferrumoxytol (FO), ferric derisomaltose (FDI), and ferric carboxymaltose (FCM) [88]. Although these intravenous preparations have the same structure, the size of the core and density of the surrounding carbohydrate significantly differ from each other. The latter three (FO, FDI, and FCM) are newer generation products with pharmacokinetic parameters allowing for a single, high-dose iron administration without the need for a test dose. Several studies have revealed that FCM is the most successful treatment for iron deficiency and iron-deficiency-related anemia [87,89,90,91].

Because increased levels of iron promote bacterial growth in vitro, there is a significant concern about the risk of infection by using iron with critically ill patients. In a multicenter, interventional study, the results revealed that intravenous iron infusion did not increase non-transferrin-bound iron, lipid, or protein oxidation in patients with anemia compared with volunteers. Conversely, iron administration decreased antioxidant levels, compatible with higher oxidative stress, in volunteers compared to in critically ill patients [57]. Moreover, patients with cardiac failure showed improved functional capacity and quality of life following intravenous FCM, and had no increase in infection [90]. Based on evidence of the efficacy and safety of available iron formulations, a systematic review suggested that currently available intravenous iron preparations are safe and effective, and FCM seems to be more beneficial for iron-deficient patients [87]. Therefore, during the recovery phase of septic patients with iron deficiency, considering iron dysmetabolism may be of substantial therapeutic benefit in improving functional recovery with the right type of drug, the best route, and the optimal timing of administration. Intravenous iron formulations used with critically ill patients are summarized in Table 1.

For the optimal timing of iron therapy, precise diagnosis of iron deficiency in critically ill patients during convalescence is important. Since the tests used for the screening of iron deficiency, such as ferritin, transferrin, and TSAT are confounded by the presence of inflammation, iron deficiency diagnosis is challenging. Recently, serum hepcidin levels have been used for the diagnosis and management of anemia in critically ill patients. It was confirmed that, if iron deficiency were diagnosed based on low hepcidin concentration (<20 ng/l) at ICU discharge, there would be a significant association with increased one-year mortality [48]. In addition, hepcidin levels would accurately guide the treatment of AID or FID in critically ill anemic patients after a prolonged ICU stay, and affect post-ICU outcomes [92]. Furthermore, a prospective observational study demonstrated that serum hepcidin concentration can be used to predict the responsiveness to iron therapy in critically ill patients with anemia, and intravenous iron supplementation can decrease the RBC transfusion requirement [93]. Therefore, it is recommended that serum hepcidin may provide a better marker of iron deficiency than the routine biochemical tests in future use.

**Table 1:** Summary of different intravenous iron formulations used with critically ill patients.

Intravenous Drug	Dosage	Outcome	Refs
IS	3, 5, 7mg/kg single infusion different dose regimens	Serum iron ↑ Hemoglobin ↑ Transferrin saturation index ↑	[94]
IS	100 mg single infusion	Oxidative stress ↓	[57]
IS	100 mg 3 times/week	Improved response to rHuEPO	[95]
IS	100 mg 3 times/week	Serum Ferritin ↑	[62]
IS	200 mg weekly	NT-proBNP, CRP ↓	[96]
FCM	500 mg, single infusion	RBC transfusion ↓	[93]
FCM	1000 mg, single infusion	Hospital readmissions at 90 days post-ICU discharge ↓	[97]
FCM	500 mg/4 days	Hemoglobin ↑	[98]
FCM	1000 mg, single infusion	90 day mortality ↓ 1 year survival ↑	[92]
FCM	equivalent to 200 mg of iron, weekly	improved 6-minute walk test and quality of life	[90]
Iron saccharate	20 mg/day	Reticulocyte count ↑ Serum transferrin receptor ↑	[60]
Iron saccharate	100 mg, 2–3 times/week	Haematocrit, Hemoglobin ↑	[99,100]
FDI	20 mg/kg, Maximum of 2000 mg, single infusion	Risk of hospital admission for heart failure and cardiovascular death ↓	[101]
FDI	1000 mg, single infusion	Cardiovascular adverse events ↓	[102]
FDI	20 mg/kg, single infusion	Hemoglobin, Ferritin, TSAT ↑	[103]
FDI	1000 mg, single infusion	Hepcidin ↑ RBC transfusion ↓	[104]
SFG	125 mg/day	No difference in 6-minute walk test	[105]
SFG	62.5 mg, 2 times/week	Haematocrit, Hemoglobin ↑	[100]

Note: IS, iron sucrose; FCM, ferric carboxymaltose; FDI, ferric derisomaltose; SFG, sodium ferric gluconate; rHuEPO, recombinant human erythropoietin; NT-proBNP, NT-pro-brain natriuretic peptide; CRP, C-reactive protein; ↑=increase; ↓= decrease.

## 7. Conclusions

In summary, critical illness, especially sepsis, can exacerbate pre-existing iron deficits since iron absorption and recycling is reduced, and stored iron is less accessible for use. Iron deficiency is common in the recovery phase of septic and COVID patients. Although low levels of serum iron may be beneficial in the early phase of critically ill patients with sepsis, persistent iron deficiency may result in iron metabolism disorder, which is not conducive to the recovery of vital organ functions. Therefore, iron supplementation may substantially benefit patients with prolonged ICU stays by improving functional recovery. Recent studies have suggested that serum levels of hepcidin can accurately guide the treatment of AID and FID in critically ill patients during the prolonged ICU stage, and forecast post-ICU outcomes. However, the optimal dosing and timing of iron administration to critically ill patients require further study.

## Data Availability

Data are contained within the article.
